# Nuclear envelope expansion is crucial for proper chromosomal segregation during a closed mitosis

**DOI:** 10.1242/jcs.181560

**Published:** 2016-03-15

**Authors:** Ai Takemoto, Shigehiro A. Kawashima, Juan-Juan Li, Linda Jeffery, Kenzo Yamatsugu, Olivier Elemento, Paul Nurse

**Affiliations:** 1 Laboratory of Yeast Genetics and Cell Biology, The Rockefeller University, New York, NY 10065, USA; 2 Laboratory of Chemistry and Cell Biology, Rockefeller University, New York, NY 10065, USA; 3 Graduate School of Pharmaceutical Sciences, The University of Tokyo, Tokyo 113-0033, Japan; 4 The Francis Crick Institute, Lincoln's Inn Fields Laboratories, London NW1 2BE, UK; 5 Department of Physiology and Biophysics, Institute for Computational Biomedicine, Weill Cornell Medical College, New York, NY 10021, USA

**Keywords:** Mitosis, Chromosome segregation, Nuclear envelope expansion, Fatty acid synthase

## Abstract

Here, we screened a 10,371 library of diverse molecules using a drug-sensitive fission yeast strain to identify compounds which cause defects in chromosome segregation during mitosis. We identified a phosphorium-ylide-based compound Cutin-1 which inhibits nuclear envelope expansion and nuclear elongation during the closed mitosis of fission yeast, and showed that its target is the β-subunit of fatty acid synthase. A point mutation in the dehydratase domain of Fas1 conferred *in vivo* and *in vitro* resistance to Cutin-1. Time-lapse photomicrography showed that the bulk of the chromosomes were only transiently separated during mitosis, and nucleoli separation was defective. Subsequently sister chromatids re-associated leading to chromosomal mis-segregation. These segregation defects were reduced when the nuclear volume was increased and were increased when the nuclear volume was reduced. We propose that there needs to be sufficient nuclear volume to allow the nuclear elongation necessary during a closed mitosis to take place for proper chromosome segregation, and that inhibition of fatty acid synthase compromises nuclear elongation and leads to defects in chromosomal segregation.

## Abstract (precis)

**Summary:** Identification of a new fatty acid synthase inhibitor for nuclear division by a chemical genetic screen revealed a link between nuclear envelope expansion and faithful chromosome segregation in a closed mitosis.

## INTRODUCTION

Genes required for the onset and progression through mitosis have been identified by screens for conditional mutants that are unable to complete cell division. The two yeasts, *Saccharomyces cerevisiae* (budding yeast) and *Schizosaccharomyces pombe* (fission yeast), have been extensively used for such studies. The first screens were for temperature-sensitive cdc (cell division cycle) mutants in the budding yeast (Culotti and Hartwell, 1971; Hartwell, 1971), which identified genes required for mitosis. Similar cdc mutants have also been identified in the fission yeast (Nurse et al., 1976), which delay onset of mitosis, as well as wee mutants which advance onset of mitosis (Nurse, 1975). A network of genes regulating the activity of the G2–mitosis cyclin-dependent kinase (Cdc2), such as cyclin B (Cdc13), Wee1 protein kinase, and the Cdc25 protein phosphatase, has been identified in fission yeast (for a review, see Nurse, 1990). Genes required for progression through mitosis have been primarily identified in fission yeast through genetic screens for cut (cell untimely torn) mutants, resulting in an un-coordinated mitosis in which the nucleus does not divide but the cell is divided by the septum cutting across the nucleus (Hirano et al., 1986). The characterization and analysis of cut mutants has revealed molecules that are important for chromosome condensation, sister-chromatid separation, anaphase-promoting proteolysis and fatty acid metabolism, as well as other processes (Yanagida, 1998).

Genetic approaches for the study of a rapid process, such as mitosis, have their limitations. The speed of action switching off a gene function can be slow, and appropriate conditional mutations must be available given that the majority of genes involved are essential. An alternative approach is to use chemical inhibitors because their speed of action is usually fast, within minutes or even seconds. However, at the present time the diversity of chemical inhibitors of mitosis is very limited compared with genetic mutants. The classical group of mitotic inhibitors consists of chemicals targeting the microtubules of the mitotic spindle, such as nocodazole and taxol (paclitaxel). They can bind tubulin and induce the disruption of microtubule dynamics, which results in kinetochores becoming detached from microtubules and activation of the spindle assembly checkpoint (Rieder et al., 1994). Proteasome inhibitors, such as MG132 and Velcade (bortezomib), prevent the metaphase–anaphase transition by inhibiting degradation of securin and cyclin B (Kawashima et al., 2012, 2013; Takeda et al., 2011; Tatebe and Yanagida, 2000). There are protein kinase inhibitors targeting cyclin-dependent kinases (CDKs) (Gray et al., 1999), Aurora kinase (Ditchfield et al., 2003; Girdler et al., 2006; Harrington et al., 2004; Hauf et al., 2003) and polo-like kinase (Steegmaier et al., 2007). The inhibitor of the kinesin Eg5 (also known as KIF11) Monastrol targets chromosome segregation (Mayer et al., 1999), and etoposide targets topoisomerase II (Baldwin and Osheroff, 2005). Unfortunately such drugs are of limited use in the fission yeast because it is highly multi-drug resistant (Kawashima et al., 2012).

To deal with the problem of multi-drug resistance and facilitate the identification of new chemical inhibitors of mitosis, we have developed a drug-sensitive fission yeast strain (MDR-sup), in which seven multi drug-resistant related genes are inactivated (Aoi et al., 2014; Kawashima et al., 2012), and have used this strain for drug screens (Kawashima et al., 2013). In this study, we used this strain to screen for chemical compounds that result in chromosome mis-segregation and have identified a new inhibitor Cutin-1. This compound restricts nuclear expansion during the closed mitosis of fission yeast, and by using genetic approaches we have shown that the target of Cutin-1 is Fas1, a subunit of fatty acid synthase (FAS).

## RESULTS

### Identification of Cutin-1 in a high-throughput screen

**Fig. 1.**
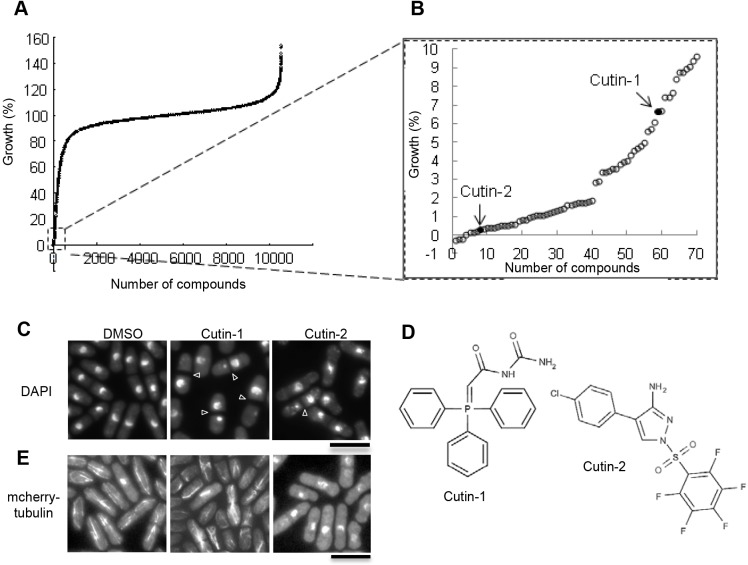
**Identification of Cutin-1.** (A) Growth assay screen using the MDR-sup strain and 2 µM of 10,371 compounds was performed by measuring the optical density (OD) of cultured cells for 17 h at 29°C in 384-well plates. Growth was normalized to the optical density values of the cell cultures without the compound to calculate relative growth rates (growth percentage). (B) A group of compounds (*n*=70) causing a growth rate of less than 10% is shown in B. Arrows show growth in the presence of two compounds, Cutin-1 and Cutin-2 which showed a mitotic *cut* phenotype. (C) Fixed and DAPI-stained cells after 6 h-incubation at 29°C with DMSO or compounds (20 µM), in the secondary screen are shown. Cutin-1 and Cutin-2 showed *cut* phenotypes in 5.6% and 10.3% of septated cells. Arrowheads show abnormal mitotic chromosome segregation with a *cut* phenotype*.* (D) Chemical structures of Cutin-1 and Cutin-2. (E) Cells expressing mCherry–Atb2 (tubulin) were treated with DMSO, 100 µM Cutin-1 for 285 min, or 1 µM Cutin-2 for 30 min followed by microscopic observation. Images of mCherry–Atb2. Scale bars: 10 µm.

**Fig. 2.**
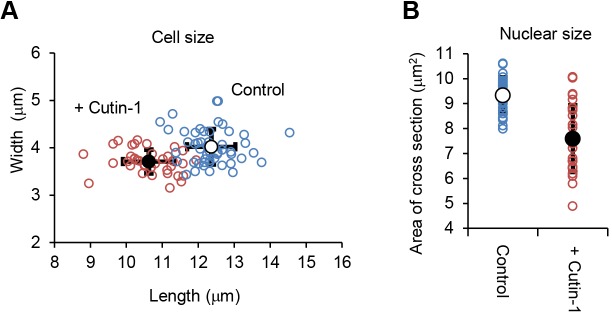
**Cutin-1 treatment reduced the size of cell and nucleus.** (A) Cell size of the septated MDR-sup cells treated with DMSO (control, blue circles) or 30 µM Cutin-1 (+Cutin-1, red circles) for 6 h at 29°C (length µm, *x*-axis; width µm, *y*-axis) were measured in DIC images using ImageJ. The values of length and width were 12.4±0.67 µm and 4.02±0.37 µm in control cells (*n*=50), and 10.6±0.72 µm and 3.71±0.25 µm in Cutin-1-treated cells (mean±s.d.; *n*=37). (B) Nuclear size was measured using the cut11-GFP expressing MDR-sup strain cultured in the same condition as (A). The sum of the two nuclear areas of maximum cross section in binucleated septated cells measured in GFP images using ImageJ were 9.33±0.72 µm^2^ in control cells (*n*=28) and 7.60±1.34 µm^2^ in Cutin-1 treated cells (*n*=28) (mean±s.d.).

Using the drug-sensitive fission yeast strain MDR-sup (Kawashima et al., 2012), we carried out a chemical screen to identify compounds that target proteins required for proper chromosome segregation during mitosis. We first identified compounds which inhibited the growth of the MDR-sup cells by more than 90% using a 10,371-member library of diverse drug-like compounds assembled in 384-well plates (see Materials and Methods). The MDR-sup cells were treated with the library compounds (2 μM) for 17 h at 29°C (six to seven generation times), and the optical density of each well was measured at 590 nm using a micro-plate reader. A subset of compounds (2.4%) inhibited growth of the MDR-sup cells by more than 50% (Fig. 1A), and the most toxic 70 compounds (Fig. 1B) were further screened by microscopic observation by staining DNA with 4,6-diamidino-2-phenylindole dihydrochloride (DAPI) to detect any defects in chromosome segregation during mitosis. In the secondary screen, we identified two compounds, Cutin-1 and Cutin-2, which resulted in abnormal nuclear division. We have not analyzed further any of the other compounds although some of them showed alterations in growth worthy of further investigation (Fig. 1A). Treatment of cells with Cutin-1 and Cutin-2 showed a *cut* phenotype (Fig. 1C,D), which is associated with defects in chromosome segregation, such as microtubule polymerization, sister-chromatid separation, chromosome condensation, anaphase-promoting proteolysis and fatty-acid metabolism (Niwa et al., 1998). Because drugs targeting microtubules have been well characterized, we were interested in compounds that did not affect microtubules, and so examined the status of microtubules in Cutin-1- and Cutin-2-treated cells. Microtubules were depolymerized in Cutin-2-treated cells, suggesting that this compound likely targets tubulin (Fig. 1E). Thus, we focused our attention on Cutin-1, a phosphonium-ylide-based compound (Fig. 1D) that did not affect microtubules (Fig. 1E). Microscopic examination revealed that Cutin-1 treatment resulted in mis-segregation of chromosomes containing *cut* phonotypes (Fig. 1C). Cutin-1 treatment for 6 h at 30 µM also reduced the size of cells by 14% and of nuclei, as shown by a reduction of 18.5% in the cross-sectional area of nuclei (Fig. 2A,B).

### Identifying the target of Cutin-1

**Fig. 3.**
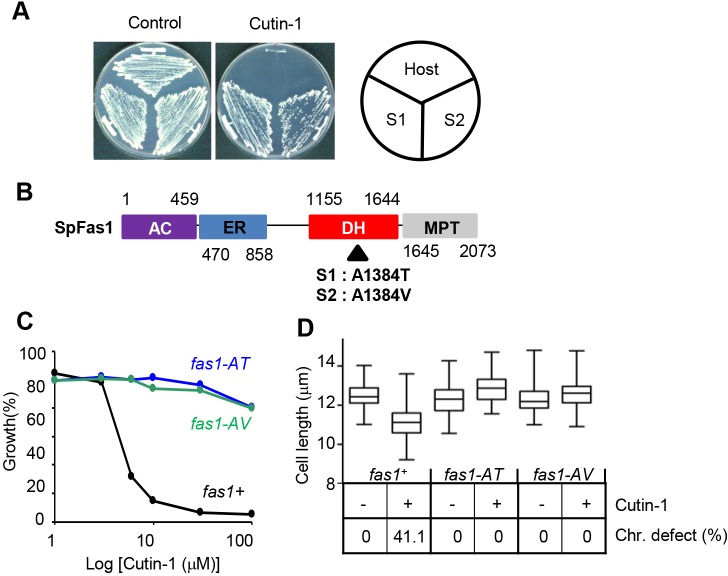
**A point mutation in *fas1* gene conferred resistance to Cutin-1.** (A) Two Cutin-1-resistant clones (S1 and S2) were isolated by mutagenesis of MDR-sup strain and selection on the Cutin-1-containing YES plates. S1 and S2 were grown on YES plates containing 10 µM Cutin-1 at 29°C. The MDR-sup strain (host) is also shown. (B) Functional domains in Fas1 and mutation sites identified by genome-wide sequencing analysis of S1 and S2 resistant clones are shown. Domains are AC, ac(et)yltransferase; ER, enoyl reductase; DH, dehydratase; and MPT, malonyl- or palmitoyl-transacylase. The S1 and S2 mutations are amino acid exchanges from alanine 1384 to threonine (A1384T) or to valine (A1384V) in the dehydratase domain of Fas1. (C) Original MDR-sup (*fas+*), and *fas1-AT-* and *fas1-AV-* introduced mutants were assayed for growth in the different concentrations of Cutin-1. (D) Cell lengths (*n*>50) and chromosome segregation defects (*n*>100) were examined in MDR-sup (*fas1+*), *fas1-AT* and *fas1-AV* mutants treated with DMSO (−) or 30 µM Cutin-1 (+) for 6 h. The graph was made using GraphPad Prism; the box represents the 25–75th percentiles, and the median is indicated. The whiskers show the minimum to maximum values.

To determine the physiological target of Cutin-1, we isolated mutants that were resistant to Cutin-1. The MDR-sup cells were chemically mutagenized, and nine resistant clones that could grow on plates containing Cutin-1 were isolated (Fig. S1A). These resistant clones could potentially contain mutations in the gene encoding the target protein of Cutin-1. Two Cutin-1-resistant strains, S1 and S2 were selected for analysis (Fig. 3A). Given that they were still sensitive to unrelated chemical inhibitors, such as brefeldin A and cycloheximide that target ER–Golgi protein transport and protein synthesis, respectively (Fig. S1B,C), we reasoned that these mutations were unlikely to be in genes involved in multi-drug resistance. After the resistant clones were back-crossed eight times with the original MDR-sup strain to reduce background mutations, common mutations between two Cutin-1-resistant strains were detected by sequencing their genomes. We identified two mutations, *fas1-A1384T* and *fas1-A1384V*, located in the *fas1* gene, which encodes the β-subunit of fatty acid synthase (FAS) (Fig. 3B; Table S1). Genetic mutants defective in fatty-acid metabolism have been previously linked to the *cut* phenotype (Saitoh et al., 1996), so we postulated that the mutation in the *fas1* gene is responsible for Cutin-1 resistance. To test this possibility, we constructed strains containing the *fas1-A1384T* or *fas1-A1384V* (hereafter *fas1-AT* and *fas1*-*AV*, respectively) mutation and found that growth inhibition in Cutin-1-treated cells was almost completely rescued by either the *fas1-AT* or *fas1-AV* mutations (Fig. 3C; Fig. S1D). The defects of cell size and chromosome segregation were also suppressed by these *fas1* mutations (Fig. 3D). These data indicate that the physiological target of Cutin-1 in fission yeast is Fas1.

### Cutin-1 targets the dehydratase domain of fatty acid synthase

FAS is an enzyme system that catalyzes fatty acid synthesis. It comprises a complex of two multifunctional proteins, an α-subunit Fas2, and a β-subunit Fas1, with an α_6_β_6_ organization in fungi. The Fas2 α-subunit contains the acyl carrier protein, ketoacyl reductase, ketoacyl synthase, and phosphopantetheine transferase activities, and the Fas1 β-subunit contains ac(et)yltransferase, enoyl reductase, dehydratase, and malonyl or palmitoyl transacylase activities (Fig. 3B) (Schweizer and Hofmann, 2004). Cerulenin is an antifungal antibiotic that inhibits FAS by binding covalently to the active center of the ketoacyl synthase domain of the Fas2 α-subunit (Flavin et al., 2010), and a point mutation within that domain confers cerulenin-resistance in budding yeast (Inokoshi et al., 1994). The mutation conferring Cutin-1-resistance in fission yeast is located in Fas1 at A1384, near the potential active center (H1361) of the dehydratase domain. Other Cutin-1-resistant clones (S3–S9) also had mutations in this domain (Fig. S1E), suggesting that Cutin-1 inhibits the dehydratase activity of FAS. Therefore, the mode of action of Cutin-1 is likely to be different from previously reported FAS inhibitors. Consistent with this interpretation, sensitivity to cerulenin is unchanged in the *fas1-AT* and *fas1-AV* mutants compared with wild-type (Fig. S1F).

**Fig. 4.**
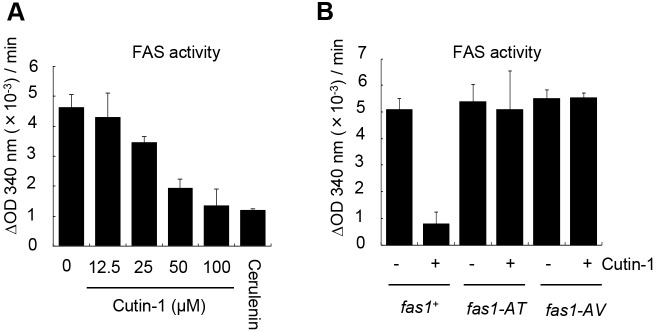
**Cutin-1 inhibits FAS activity *in vitro*.** (A) *In vitro* FAS activity was assayed by measuring the OD_340_ to detect NADPH utilization. Cutin-1 inhibits FAS activity of MDR-sup cell extracts in a dose-dependent manner. Cerulenin, a known FAS inhibitor, was used as a positive control at 10 µM. (B) *In vitro* FAS activities were compared in MDR-sup (*fas1^+^*), *fas1-AT*, and *fas1-AV* strains with or without Cutin-1 (100 µM). FAS activity of the *fas1^+^* strain was inhibited by Cutin-1 whereas the activities of the *fas1-AT* or *fas1-AV* mutants were not affected by Cutin-1. Results are mean±s.e.m. (three independent experiments).

To examine whether Cutin-1 inhibits FAS activity directly, we treated a fission yeast extract with Cutin-1 and assayed for FAS activity *in vitro* (Lynen, 1969; Niwa et al., 1998). We found that Cutin-1 inhibited FAS activity in a dose-dependent manner *in vitro* (Fig. 4A). Inhibition of FAS activity by Cutin-1 was completely suppressed when extracts from the *fas1-AT* or the *fas1-AV* mutants (Fig. 4B) were used, indicating that Cutin-1 directly inhibits FAS activity and does so through the dehydratase domain of Fas1.

### Cutin-1 treatment suppresses FAS-mediated nuclear envelope expansion

**Fig. 5.**
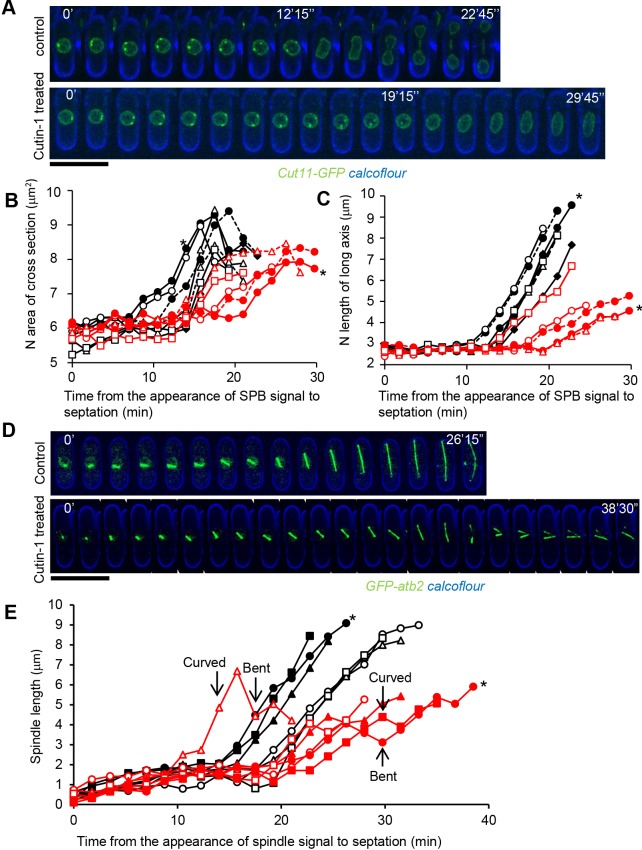
**Cutin-1 suppressed FAS-mediated nuclear expansion in mitosis.** (A) Time-lapse images with 105-s intervals of cells expressing Cut11–GFP (green) treated with or without Cutin-1 (100 µM). Cell walls were visualized with Calcoflour (blue). (B) The nuclear surface area of the maximum cross section of Cut11–GFP signal at each time-point image was measured. Black markers show control and red markers show Cutin-1-treated cells. Asterisks indicate the measurements of cells shown in A. (C) The nuclear lengths along the long axis were measured in the same images as for B. (D) Time-lapse images of cells expressing Atb2–GFP (tubulin; green) for spindles and Calcoflour (blue) with or without Cutin-1. (E) Spindle lengths are plotted. Black markers are control cells and red markers are Cutin-1-treated cells. Scale bars: 10 µm.

Given that FAS is required for nuclear envelope expansion during mitosis in fission yeast (Yam et al., 2011), we visualized the nuclear envelope using a GFP-fused Cut11-expressing strain, and observed changes of nuclear envelope and nuclear size during mitosis in Cutin-1-treated cells by time-lapse photomicrography. Nuclear size was estimated by measuring the area of the maximum cross section of nuclei in time-lapse images of Cut11–GFP. Nuclear size increased during mitosis in untreated control cells, whereas this increase was suppressed in Cutin-1-treated cells (Fig. 5A,B), consistent with the phenotypes observed in cells treated with cerulenin (Yam et al., 2011). We also measured the length of the nucleus along its longest axis in the presence and absence of Cutin-1. The nucleus extended substantially during mitosis but this extension was inhibited in Cutin-1-treated cells (Fig. 5C), indicating that FAS-mediated nuclear envelope expansion increases the long axis of the nucleus. Given these results, we propose that increases in the volume and the long axis of the nucleus are required for proper segregation of chromosomes. We also observed that the presence of Cut11–GFP at the spindle pole body (SPB) (West et al., 1998) was retained for a longer period in Cutin-1-treated cells (16.5±2.0 min) compared with control cells (12.5±1.2 min) (mean±s.d., *n*=7; Fig. 5A,B), suggesting that the metaphase–anaphase transition was slightly delayed in Cutin-1-treated cells. Both the defect in nuclear envelope expansion and the delay in the metaphase–anaphase transition were suppressed by the *fas1-AT* mutation (Fig. S2A,B), indicating that these phenotypes are unlikely due to off-target effects by Cutin-1. We examined spindle dynamics in Cutin-1-treated cells by visualizing microtubules using a GFP-fused Atb2-expressing strain (encoding α-tubulin), and found that the spindles became curved, buckled and bent during anaphase (Fig. 5D,E). These phenotypes were also suppressed in the *fas1-AT* mutant (Fig. S2C,D). These data suggest that FAS inhibition by Cutin-1 causes the spindle to buckle due to the slow expansion of the nucleus putting pressure on the elongating spindle.

### Cutin-1 treatment inhibits nucleolus segregation

**Fig. 6.**
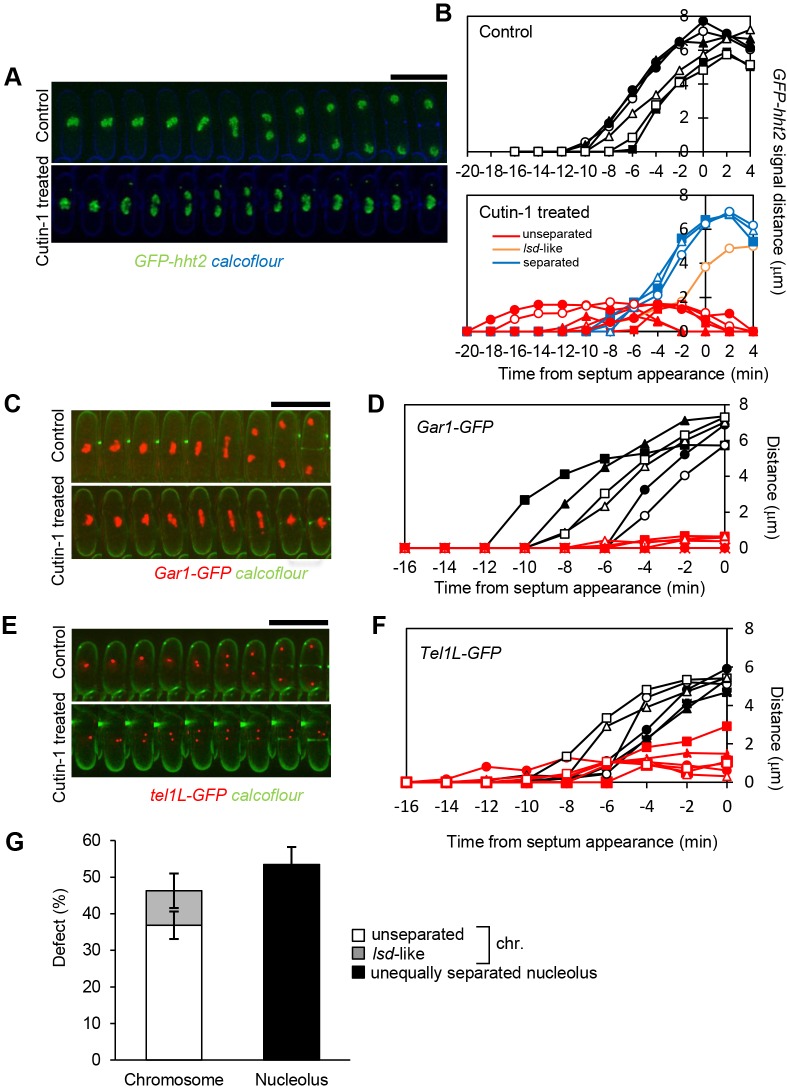
**Nucleolus segregation was defective in Cutin-1-treated cells.** (A) Time-lapse images with 105-s intervals of cells expressing Hht2–GFP (histone; green) treated with or without Cutin-1. Cell walls were visualized with Calcoflour (blue). Conditions are the same as in Fig. 5. The chromosomes in Cutin-1-treated cells typically separate and then fuse back together. (B) The distances of Hht2–GFP signals were measured from time-lapse images and plotted. Control (black in upper graph) and Cutin-1-treated cells (separated, blue; *lsd*-like, orange; and unseparated, red in lower graph) are plotted. Data for cells in A are shown as filled circles. (C) Time-lapse images with 2-min intervals of cells expressing Gar1–GFP (nucleolus, red) treated with or without Cutin-1. Cell walls were visualized with Calcoflour (green). (D) The distances of Gar1–GFP signals were measured from time-lapse images and plotted. Control and Cutin-1-treated cells are plotted as black and red, respectively. Data for cells in C are shown as filled circles. (E) Time-lapse images with 2-min intervals of cells expressing Tel1–GFP (telomere of the chromosome 1, red) treated with or without Cutin-1. Cell walls were visualized with Calcoflour (green). (F) The distance between the telomere dots in cells treated with (red) or without (black) Cutin-1. Data for cells in E are shown as filled circles. (F) The rate of mitotic chromosome defects and nucleolus segregation defects in septated cells treated with 30 µM Cutin-1 for 5 h followed by fixation. Chromosomal DNA is visualized by DAPI stain and the nucleolus by Gar1–GFP signal. Results are the mean±s.d. (%) with the defect indicated for cells in each of five photo images (containing more than 40 cells). Scale bars: 10 µm.

To examine how chromosomes are mis-segreagated in Cutin-1-treated cells, we visualized the chromosomes using GFP-fused histone H3 (Hht2), and recorded the movement of the chromosomes from metaphase to septum formation by time-lapse microscopy. In control cells, chromosomes were equally segregated and almost reached the edges of the cell by the time the septum was formed (Fig. 6A,B, upper panels). In 13 out of 25 Cutin-1-treated cells, chromosomes started to segregate as in control cells, but then failed to separate further and subsequently re-associated, probably due to collapse of the spindle. This was followed by septum formation, accounting for the observed chromosomal mis-segregation *cut* phenotype (Fig. 6A,B, lower panels). In the remaining (12 out of 25) Cutin-1-treated cells, the chromosomes segregated normally, but four of these cells generated large and small daughters (*lsd*)-like phenotypes (Saitoh et al., 1996). We speculated that the re-association of segregated chromosomes in Cutin-1-treated cells might be the consequence of continued linkage between sister chromatids. To examine whether separation of the telomere and rDNA or nucleolus regions was impaired, we visualized the telomere region of chromosome I using a strain in which the *tel1L* region was marked by GFP, and the nucleolus marked by a GFP-fused nucleolus protein Gar1 (Tada et al., 2011). Time-lapse images of Cutin-1-treated cells showed that some nucleoli remained unseparated in the middle of cells, which resulted in the *cut* phenotype, whereas the nucleolus in the control cells separated before septum formation (Fig. 6C,D). The telomeres initially separated in a similar way in both the control cells and the Cutin-1-treated cells, but subsequent further separation was inhibited in the Cutin-1-treated cells (Fig. 6E,F), probably due to the nucleolus remaining unseparated. Consistent with these results, the defect in nucleolus separation defect induced by Cutin-1 treatment was greater than that of chromosome mis-segregation (Fig. 6G). Given this data, we suggest that nucleolus and rDNA separation is sensitive to reduced nuclear envelope expansion and might be a major cause of the mitotic chromosome defects following Cutin-1 treatment.

### Chromosome segregation requires adequate nuclear elongation during a closed mitosis

**Fig. 7.**
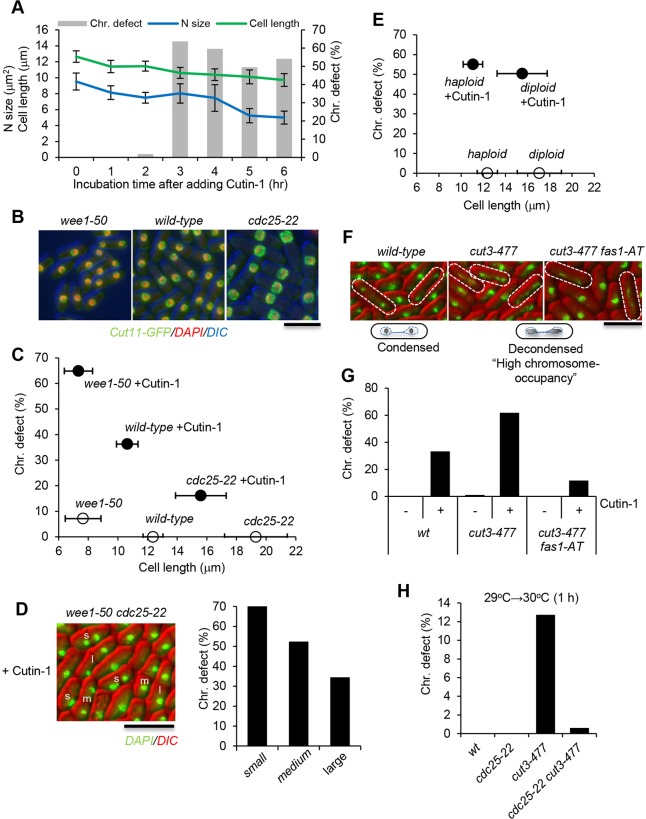
**Sufficient nuclear volume is required for proper chromosome segregation.** (A) The timing of mitotic chromosome defects and cell and the nuclear size over time from 100 µM Cutin-1 addition were analyzed using a Cut11–GFP-expressing MDR-sup strain. The percentage (%) of cells showing a chromosome defects (*n*>200) as observed in DAPI images, the nuclear surface area of maximum cross section (µm^2^, *n*>40) in GFP images, and the cell length of the long axis (µm, *n*>50) in DIC images of septated cells were measured using ImageJ. Results are mean±s.d. (B) The *wee1-50*, wild-type, and the *cdc25-22* cells expressing Cut11–GFP were cultured at 29°C, fixed with methanol and stained with DAPI. Images show Cut11–GFP (green), DAPI (red) and DIC (blue). (C) The percentage (%) of cells showing mitotic chromosome defects (*y*-axis, *n*>100) and the cell lengths (µm in *x*-axis) of each strain after incubation for 6 h at 29°C with DMSO (white circles) or with 30 µM of Cutin-1 (black circles). Septated cells were counted. Wild-type, *cdc25-22*, and *wee1-50* all have the MDR-sup background. Results are means (±s.d. for cell length) (*n*>50). (D) The *wee1-50 cdc25-22* double mutant was treated with Cutin-1, fixed and DAPI-stained (same conditions as in C). DAPI (green) and DIC images (red) are shown in the left panel. About 90% of wild-type cells treated with Cutin-1 are between 9.5 µm and 11.5 µm in length, thus we defined cells in this range as ‘medium’; cells less than 9.5 µm in length were defined as ‘small’, and cells more than 11.5 µm were defined as ‘large’. Small cells (s), wild-type medium size cells (m), and large cells (l) that have septa are indicated. The percentage (%) of cells showing mitotic chromosome defects in septated cells of *wee1-50 cdc25-22* double mutants treated with 30 µM Cutin-1 for 6 h are shown for the three separated groups. (E) The percentage (%) of cells showing mitotic chromosome defects (*y*-axis, *n*>300) and the cell lengths (µm in x-axis) of diploid and control haploid strains treated with DMSO (white circles) or with 30 µM of Cutin-1 (black circles) (same conditions as C). Results are means (±s.d. for cell length) (*n*>50). (F) Fixed and DAPI-stained wild-type and *cut3-477* cells incubated at 29°C. DAPI (green) and DIC images (red) are shown. Chromosomes are less condensed and highly occupied with larger volume in nuclei in *cut3-477* cells compared to wild-type cells. (G) The percentage (%) of cells showing chromosome segregation defects in septated cells of wild-type (wt), *cut3-477* and *cut3-477 fas1-AT* strains after 6 h-incubation with DMSO (–) or 30 µM Cutin-1 (+). (H) The percentage (%) of cells showing chromosome defects observed in *cut3-477* cells cultured at 29°C and shifted up to 30°C for 1 h, were suppressed by increasing the nuclear volume using a strain with the *cdc25-22* mutation. Scale bars: 10 µm.

Based on above observation of the phenotypes in Cutin-1-treated cells, we hypothesized that adequate nuclear elongation by FAS-mediated nuclear envelope expansion is an important prerequisite for proper chromosome segregation in fission yeast. Supporting this hypothesis, the chromosome segregation defects induced by Cutin-1 only appeared at around 3 h whereas nuclear and cell size reductions were detected within 1 h after Cutin-1 treatment (Fig. 7A). The mitotic phenotype induced by Cutin-1 appears during the first mitosis after nuclear or cell size became reduced. The nuclear or cell size ratio appears to be crucial for chromosome segregation, so to test this possibility, we used genetic mutants with differently sized nuclei. A temperature-sensitive mutant of *cdc25*, encoding a tyrosine phosphatase that activates the Cdc2 kinase, becomes arrested at the G2/M transition at the restrictive temperature (36°C), but can enter mitosis at an enlarged cell size resulting in a nucleus of increased size at the semi-permissive temperature of 29°C (Neumann and Nurse, 2007) (*cdc25-22*, Fig. 7B). Mutations in *wee1*, the protein kinase that counteracts the Cdc25 phosphatase, lead to a premature entry into mitosis, resulting in smaller cells and smaller nuclei (Neumann and Nurse, 2007) (*wee1-50*, Fig. 7B). We found that the defects in chromosome segregation caused by Cutin-1 treatment were partially suppressed in *cdc25-22* cells with enlarged nuclei, being present in 16.1% at the semi-permissive temperature (29°C) compared with 36.3% in wild-type cells (Fig. 7C). In contrast, chromosome mis-segregation in Cutin-1-treated cells was increased in *wee1-50* mutant cells to 64.9% (Fig. 7C). The double mutant *cdc25-22 wee1-50* strain showed a heterogeneous cell and nucleus size with a mixture of *cdc25-22*-like ‘large’, wild-type-like ‘medium’ and *wee1-50*-like ‘small’ cells (Fig. 7D). Cutin-1 treatment of this double mutant showed that chromosome segregation defects were inversely correlated with nuclear size (Fig. 7D). Consistent with this, *cdc25-22* mutant cells were partially rescued with respect to growth reduction by Cutin-1 treatment at the semi-permissive temperature (Fig. S4A). These results suggest that a minimum nuclear size is required for sufficient nuclear elongation to ensure faithful chromosome segregation. We also observed the chromosome segregation defects caused by Cutin-1 in diploid cells, where cell and nuclear sizes are large with twice the number of chromosomes (Neumann and Nurse, 2007). The diploid cells showed no substantial decrease in the Cutin-1-induced chromosome segregation defects compared to control haploid cells, even though their cell sizes were close to *cdc25-22* cells (Fig. 7E). From this result, we speculated that more nuclear elongation would be required for completing chromosome segregation when there are more chromosomes in a nucleus and so the space occupied by chromosomes is increased. To test this possibility, we used a mutant of condensin that has a role in mitotic chromosome compaction (Saka et al. 1994). In fact, *cut3-477*, a temperature-sensitive mutant of the condensin subunit Cut3, showed decondensed mitotic chromosomes, which spread and occupied more space in a nucleus at a semi-permissive temperature compared to wild-type (Fig. 7F). We examined the mitotic chromosome defects caused by Cutin-1 in *cut3-477* cells and found that Cutin-1-treated *cut3-477* showed more defects than wild-type cells (Fig. 7G). This increase was not observed in a *fas1-AT cut3-477* double mutant (Fig. 7G). In addition, the chromosomal segregation defects found in *cut3-477* cells at a semi-permissive temperature without Cutin-1 were substantially suppressed when the nuclear volume was increased by a *cdc25-22* mutation in a wild-type *fas1^+^* background (Fig. 7H). These results suggest that the regulations of the volume of both a nucleus and chromosomes might contribute to the proper chromosome segregation in a synergistic manner during a closed mitosis.

## DISCUSSION

In this study, we have used a multi-drug-sensitive fission yeast strain and identified a new inhibitor of mitosis and chromosome segregation by a chemical screen, which mimics successful genetic screens using fission yeast (Hirano et al., 1986). One of the hit compounds, Cutin-1, targets the Fas1 subunit of FAS and its treatment induced *cut* phenotypes, consistent with previous reports that a *fas2/lsd1-H518′* temperature-sensitive mutant shows mitotic defects (Saitoh et al., 1996). Based on our genetic and biochemical evidence, Cutin-1 targets the dehydratase domain in Fas1 subunit, whereas cerulenin, an antifungal antibiotic, targets the ketoacyl synthase domain in Fas2 subunit. Therefore, the mode-of-action of Cutin-1 is different from cerulenin. Given that previous genetic screens have identified not only the FAS gene, but also other genes that are important for chromosome condensation, sister-chromatid separation and anaphase-promoting proteolysis (Yanagida, 1998), further large-scale chemical screens would be expected to identify compounds that target such important factors.

During anaphase, FAS activity is essential for nuclear envelope expansion (Yam et al., 2011). Our time-lapse microscopic experiments show that nuclear volume and length are rapidly increased during anaphase, probably followed by nuclear envelope expansion. Given that the larger nucleus in a *cdc25-22* strain suppressed the chromosome segregation defects caused by Cutin-1 treatment and the smaller nucleus in a *wee1-50* strain led to more severe defects, the rapid increase in nuclear volume and length during anaphase appear to be an important function of FAS to bring about proper chromosome segregation during a closed mitosis. Detailed time-lapse microscopic observations of Cutin-1-treated cells showed that the bulk of chromosomes transiently separate, but nucleoli rarely separate (Fig. 6). This results in spindle collapse and re-association of sister chromatids, which leads to chromosome mis-segregation. These observations suggest that the tension generated by the spindle in Cutin-1-treated cells is not sufficient for nucleoli separation. The persistent cohesion of the rDNA region between sister chromatids may resist the forces generated by spindle elongation, leading to spindle collapse. We cannot discriminate whether the failure to separate the nucleolus might be the consequence or the cause of chromosome segregation failure by Cutin-1 treatment, given that the nucleolus is separated after most of the chromosome regions containing telomeres are separated. In *S. cerevisiae*, nuclear envelope expansion occurs in a specific region close to nucleolus when cells are arrested in mitosis (Webster et al., 2010), so it is possible that FAS-mediated nuclear envelope expansion near the nucleolus also helps nucleoli segregation.

We found that increased nuclear volume and elongation is more important especially when condensin is partially inhibited (Fig. 7G,H). It has been reported that the segregation of nucleolus and rDNA regions are promoted by the condensin complex in *S. pombe* (Nakazawa et al., 2008; Tada et al., 2011), and so this function is the possible cause of the additive defects observed in the condensin mutant treated with Cutin-1. In addition to this, our data suggest that increased chromosome occupancy by decondensed chromosomes in the condensin mutant might require more nuclear expansion, thus increasing sensitivity to inhibition of FAS activity. Consistent with this hypothesis, a *cdc25* mutant with an enlarged nucleus suppressed chromosome segregation defects in a condensin mutant (Fig. 7H), even though chromosome compaction was not effected (Fig. S3) and the reduced nuclear size in a *wee1* mutant strain showed synthetic temperature sensitivity with the condensin mutant (Fig. S4B). Although the major phenotype in Cutin-1-treated cells is mis-segregation of chromosomes, some cells showed an *lsd*-like phenotype (Fig. 6G). Therefore, as reported previously (Saitoh et al., 1996), other nuclear components in addition to the nucleoli might also be mis-segregated by Cutin-1 treatment.

In this proof-of-principle approach described here, we demonstrate how new chemical compounds targeting mitosis can be isolated in fission yeast, and how their targets can be readily identified by using genetic approaches. Such compounds will provide new chemical inhibitors with potentially rapid modes of action, which will be helpful for the further study of dynamic aspects of mitosis.

## MATERIALS AND METHODS

### Fission yeast strain

All strains used are listed in Table S2. Standard growth conditions were used (Moreno et al., 1991). The construction of strains which have a MDR-sup background were performed as described previously (Kawashima et al., 2013).

### Construction of the diverse compound library

The screening libraries comprised a sub-set of 10,371 compounds chosen from a larger 175,000-member collection of commercially available compounds from The Rockefeller University High-Throughput Screening Resource Center (http://www.rockefeller.edu/htsrc/libraries). With the exception of the LOPAC and Prestwick collection of known drugs, the larger collection was chosen based on properties such as structural diversity and novelty, drug-like qualities, and stability of functional groups. The subset of 10,000 structurally diverse compounds was chosen by using Pipeline Pilot software (Accelrys, San Diego, CA) to generate a list of screening plates. The ‘sd’ file of the entire Rockefeller collection was inputted into the software to generate a list of 384-microtiter plates that contained the largest relative diversity of compounds based on functional class fingerprinting. More specifically, the ‘diverse molecules’ protocol from the Pipeline Pilot software was set to use a maximum dissimilarity algorithm based on a numeric fingerprint distance function called ‘functional class fingerprinting’ which was set to use four adjacent bonds around each atom in a molecule as the basis for similarity scoring and clustering. In the end, the collection of 30 plates contained 2112 ChemDiv (San Diego, CA) compounds, 352 AMRI (Albany, NY) compounds, 5280 Enamine (Kiev, Ukraine) compounds, 704 Life Chemicals (Kiev, Ukraine) compounds and 2112 Chembridge (San Diego, CA) compounds. All compounds were stored as 5 mM DMSO stock solutions at −30°C in heat-sealed 384-well polypropylene low-volume microtiter plates.

### Chemical compounds

Cutin-1 (CAS Registry Number: 53296-08-5), and Cutin-2 (CAS Registry Number: 957489-42-8) were purchased from ChemDiv (San Diego, CA). Cutin-1 was also synthesized in the laboratory of Graduate School of Pharmaceutical Sciences, the University of Tokyo. Synthesized compounds in the laboratory showed the same growth inhibition and chromosome defects in the MDR-sup strain as the purchased compounds. Celurenin (Sigma-Aldrich, St Louis, MO), cyclohexiamide (Sigma-Aldrich), and Brefeldin A (LC Laboratories, Wobun, MA) were also used. They were dissolved in DMSO, kept at −20°C, and used in 0.1–1% DMSO in medium.

### Growth assay for screen and validation

The MDR-sup strain (SAK84) growing logarithmically [optical density at 595 nm (OD_595_)=0.5] was diluted 50 times for screening. The culture was incubated for 15 h at 29°C in 348-well plates with 2 µM of each compound (total volume 50 µl per well). Multidrop Combi (Thermo Scientific, Waltham, MA) was used to dispense the cells into wells of 384-well plates (clear flat-bottom PS plate from Greiner, Monroe, NC). Growth was measured by a microtiter plate reader (EnVision, 590-nm filter, PerkinElmer, Waltham, MA). The growth ratio was calculated by dividing OD values by that of a control well incubated with DMSO (Kawashima et al., 2012). In a validation growth assay, logarithmically growing cells were diluted to OD=0.1 and its 10 times dilution were used for assays. The cell culture (1 ml) mixed with compound at several dilution series was incubated for 15 h at 29°C. The concentration of DMSO was usually 0.1% in assay cultures. The optical density was measured to calculate the growth ratio.

### Microscopic observation

For methanol fixation, a cell pellet from a 1 ml culture was suspended in 1 ml chilled methanol and incubated for >12 h at −20°C. The fixed cells were mixed with DAPI and the cell pellets dissolved in PEMS (100 mM PIPES, pH 6.9, 1 mM EGTA, 1 mM MgSO_4_ and 1 M sorbitol). Images were acquired at room temperature on a microscope (Axioplan2 or Axio Imager 2, Carl Zeiss, Oberkochen, Germany), equipped with a CoolsnapHQ camera (Roper Scientific, München, Germany), and were processed with MetaMorph software (Molecular Devices, Sunnyvale, CA, USA) or Axio Vision 4.8 software (Carl Zeiss). A *z*-stack of ∼3 µm thickness with 0.3-µm pitch planes was captured and combined in a single image. Cell size was measured by using the differential interference contrast (DIC) image. Nuclear size was measured by using Cut11–GFP signals. For time-lapse images of cells treated with Cutin-1, we precultured cells in YE medium containing 100 µM Cutin-1 for 3 h (over one cell cycle) at 32°C and transferred cells to a microfluidic cell culture system (CellASIC, ONIX, Merck Millipore, Darmstadt, Germany) equipped with a DeltaVision imaging system and acquired *z*-stack images in each time point with the correct interval for 105 or 120 min at 30°C.

### Isolation of Cutin-1-resistant mutants

MDR-sup cells were mutagenized by treatment with 1-methyl-3-nitro-1-nitrosoguanidine (NTG) (50 µg/ml) in TM buffer [50 mM Tris-HCl, 50 mM maleic acid, 7.5 mM (NH_4_)_2_SO_4_, 0.4 mM MgSO_4_·7H_2_O, pH 6.0] for 30 min, incubated in YE medium for >3 h, and spread onto plates containing 15 µM Cutin-1. Nine Cutin-1-resistant mutants were isolated from ∼10^6^ cells.

### Analysis of the whole-genome sequencing data

63 M and 42 M 51-bp-long single end reads were obtained for clone S1 and S2, respectively. Reads were aligned to the reference *S. pombe* genome (downloaded on 4 February 2011) using BWA (Li and Durbin, 2009) with default parameters. 84% and 85% of the reads mapped unambiguously and were retained for further analysis. Clonal reads (i.e. reads mapping at the same genomic position and on the same strand) were collapsed and replaced by the read with highest average quality score. Single-nucleotide variant (SNV) detection was performed using the following approach. For each nucleotide in the *S. pombe* genome, we calculated the number of overlapping reads and determined which of these reads showed a mismatch at that position compared to the reference genome. Only positions with at least four overlapping reads were further considered. Assuming that the large majority of mismatches between the reads and the reference genome are due to sequencing errors, we determined the probability of observing a given number of mismatches at a given genomic position by chance, given the overall error rate of the experiment. Assuming that mismatch counts follow a binomial distribution, this probability is calculated by:
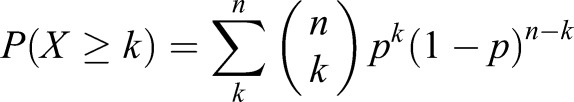


where *n* represents the total number of reads overlapping with the considered position, *k* represents the number of these reads that contain a mismatch at that genomic position, and *P* is the overall mismatch rate in the entire set of aligned reads. These probabilities were adjusted for multiple hypotheses testing using the Benjamini–Hochberg approach, and a false discovery rate of 1% was used for SNV calling. All detected SNVs were then annotated using a custom script that finds overlapping *S. pombe* genes (based on Sanger annotation downloaded from ftp://ftp.sanger.ac.uk/pub/yeast/pombe/GFF/), determines whether the variant leads to missense, nonsense, synonymous, 3′UTR, 5′UTR changes, and calculate BLOSUM62 scores for missense variants. All scripts and programs used for this analysis can be found at http://icb.med.cornell.edu/wiki/index.php/Elementolab/TargetID.

### *In vitro* fatty acid synthase assay

We followed the method of *in vitro* fatty acid synthase assay described previously (Lynen, 1969; Niwa et al., 1998). Logarithmically growing cells were collected by centrifugation (800 ***g*** for 2 min), rinsed with H_2_O twice, and the aliquots of cell pellet (−50 µl) can be stored at −80°C for the assay. The cell pellet was suspended in the same volume of buffer A [50 mM Tris-HCl pH 8, 10% glycerol, 1 mM EDTA, 1 mM DTT, 10 mM PMSF, 0.2 M NaCl, 1× protease inhibitor complete cocktail (GE Healthcare Life Sciences, Pittsburgh, PA)], transferred to a screw-lid tube supplied with 1.2 ml grass beads on ice, and then vortex mixed with FastPrep instrument [three times for 20 s each time with 30-s intervals, using input 4 of a FP120 machine (BIO101/Savant, Carlsbad, CA)] at 4°C. A similar volume of buffer A was added to the tube. Then, the bottom of the tube was pierced with a needle to make a hole, and the tube was set on a new tube and spun down at 2500 ***g*** for 1 min. The supernatant was transferred to a new tube and centrifuged at 16,000 ***g*** for 10 min twice. The cell extract of supernatant was used for the *in vitro* Fas assay. For a 500 µl assay, 10 µl of cell extract (87 µg total protein) in buffer A containing the inhibitor or not was pre-incubated at 25°C for 30 min and 490 µl of assay buffer was added (concentration in 500 µl assay: 100 mM potassium phosphate pH 6.6, 2.5 mM EDTA, 1.5 mM NADPH, 0.6 mM acetyl-CoA and 0.3 mg/ml BSA). Adding 10 µl of 7 mM malonyl-CoA to the prepared mixture starts the reaction and the assay tube was incubated at 25°C for 60 min. Before addition of malonyl-CoA and 60 min after the addition, the optical density at 340_ _nm (OD_340_), corresponding to the NADPH concentration was measured in silica cuvettes. The consumption of NADPH by fatty acid synthase was calculated from the difference in the values before and after incubation.
